# Costs and quality of life for prehabilitation and early rehabilitation after surgery of the lumbar spine

**DOI:** 10.1186/1472-6963-8-209

**Published:** 2008-10-09

**Authors:** Per Rotbøll Nielsen, Jakob Andreasen, Mikael Asmussen, Hanne Tønnesen

**Affiliations:** 1 Centre of Head and Orthopaedics, Department of Anaesthesiology, Rigshospitalet, University of Copenhagen, Denmark; 2 Clinical Unit of Health Promotion/WHO Collaborating Centre for Evidence-Based Health Promotion, Bispebjerg Hospital, University of Copenhagen, Denmark; 3 Research Unit for Technical Health Assessment, Hvidovre Hospital, University of Copenhagen, Denmark; 4 Copenhagen Business School, Copenhagen, Denmark

## Abstract

During the recent years improved operation techniques and administrative procedures have been developed for early rehabilitation. At the same time preoperative lifestyle intervention (prehabilitation) has revealed a large potential for additional risk reduction.

The aim was to assess the quality of life and to estimate the cost-effectiveness of standard care versus an integrated programme including prehabilitation and early rehabilitation.

**Methods:**

The analyses were based on the results from 60 patients undergoing lumbar fusion for degenerative lumbar disease; 28 patients were randomised to the integrated programme and 32 to the standard care programme.

Data on cost and health related quality of life was collected preoperatively, during hospitalisation and postoperatively. The cost was estimated from multiplication of the resource consumption and price per unit.

**Results:**

Overall there was no difference in health related quality of life scores. The patients from the integrated programme obtained their postoperative milestones sooner, returned to work and soaked less primary care after discharge. The integrated programme was 1,625€ (direct costs 494€ + indirect costs 1,131€) less costly per patient compared to the standard care programme.

**Conclusion:**

The integrated programme of prehabilitation and early rehabilitation in spine surgery is more cost-effective compared to standard care programme alone.

## Background

During the recent years improved surgical and anaesthesiological techniques as well as administrative procedures have been developed for fast tracking surgery. This programme describes a multi-modal approach, which includes optimal operation techniques, better pain reduction, early nutrition and aggressive postoperative mobilisation [1]. An important part of the program is to inform and prepare the patients for the accelerated pathway. The results have been promising, especially regarding the economical consequences of the shortened length of stay and rehabilitation[2].

In the same time randomised clinical studies have revealed a large potential for effect of adding preoperative lifestyle intervention (prehabilitation) to conventional surgical procedures. A 6–8 weeks programme of preoperative smoking cessation intervention halved the complication rate after hip- and knee replacement[3], and a 4 weeks programme of preoperative alcohol abstinence intervention halved the development of all kinds of complications in harmful drinkers after colorectal resection[4]. Furthermore, some studies of preoperative physical training showed a beneficial effect on the length of stay and complication rate, while others reported no effect at all [5,6]. Hitherto, prehabilitation has not been shown to worsen the postoperative outcome on a randomised trial.

The aim of the present study was to compare the economic impact and quality of life of surgery for degenerative lumbar spine disease with and without integration of prehabilitation and early rehabilitation.

## Methods

The present study included data collected in a randomised controlled trial of 60 patients undergoing lumbar fusion for degenerative lumbar disease[7]. After informed consent 28 patients were randomised to an integrated programme and 32 to the standard care programme. Their characteristics are given in table 1.

**Table 1:** Characteristics of the 28 patients randomised to the integrated programme, which comprehended prehabilitation and early rehabilitation, and the 32 patients randomised to the standard care programme, exclusively

**Preoperatively**	**Integrated programme n = 28**	**Routine programme n = 32**
Women	17 (61%)	19 (59%)
Age (years)	48 (31–72)	52 (31–88)
Weight (kg)	77 (48–118)	77 (51–107)
BMI (kg/m^2^)	25 (21–33)	26 (17–33)
Daily smokers	3 (11%)	5 (16%)
Harmful drinkers	0	0
ASA Classification (I–V)^a^	I (I–II)	I (I–II)
		
**Postoperatively**		

Patients with major complications ^a^	8	8
Cardiopulmonary insufficiency	0	0
Thrombo-Embolic complications	0	0
Haematomas	1	2
Severe pain (> 70 at VAS)	6	7
Allergic reaction	1	0
Patients with minor complications	4	4
Pneumonia (antibiotics)	0	0
Wound infection (antibiotics)	0	1
Urinary tract infection (>10^5 ^bacteria/ml)	2	0
Urinary retention (tubulation)	0	1
Constipation (laxities)	2	2
Patients with adverse events	3	1
Delayed order of nursing at home	2	0
Surgery on Friday ^b^	1	1

Second surgery	0	1 (3%)
Hospital stay after surgery (days)	5 (4–9)*	7 (5–15)
Stay until reaching all milestones	4 (1–6)*	6 (3–13)
Extra stay due to complications	18*	30

(Numbers and per cent or median and range)

* P < 0.01 ^a ^Requiring intensive treatment or secondary surgery ^b^Not followed by physiotherapy in the weekend

Data collection included cost and quality of life for each patient in the preoperative period under hospitalisation and in the postoperative period. The costs originated from three categories; staff resources, equipments and purely bed costs. The bed costs included salary of the nurses and porters, food, clothes, laundry and cleaning.

The preoperative programme for the intervention group involved extra resources for prehabilitation; information and instruction, optimisation of pain treatment[7] in relation to the 6-weeks home training programme of 30 minutes per day followed-up by phone calls and log-books, 6-weeks smoking cessation programme with free nicotine replacement therapy and weekly follow-up visits[3], 4-weeks alcohol abstinence programme with free medication (benzodiazepines, disulfiram and B-vitamins) and weekly follow-up visits[4]. Postoperatively, the integrated programme included double the time for physiotherapy during their stay at hospital, protein-rich drinks, balanced and patient-controlled epidural analgesia and a follow-up visits for smokers and harmful drinkers. Milestones for both groups are defined as follow: 1. Assisted positional change in bed, 2. Independent positional change in bed, 3. Assisted moblization to bedside, 4. Independent moblization to bedside, 5. Assisted moblization in walkingframe, 6. Independent moblization to walkingframe, 7. Independent personal hygiene, 8. Independent daily function on ward, 9. Walking without aids, 10. Complete training programme, 11. Independent stair climb and 12. Discharge to home.

For both groups the direct costs were identical for primary surgical intervention and postoperative activities related to the standard care programme.

Postoperatively, data for estimation of the direct costs was collected for each patient regarding length of stay and second surgery, table 1. After discharge, the contact within the first 3 postoperatively months to general practitioners, emergency wards and to physiotherapists was registered. Data collection for indirect costs involved loss of productivity until return to work.

Quality of life was assessed by self-reports. The patients filled in the generic Quality of life survey tool 15D at six different time points (at inclusion, at the day of surgery, at the day of discharge and 1,3 and 6 months postoperatively[8]).

### Analyses

The costs were estimated from multiplication of the resource consumption and unit costs. The focus was on the differences between the two programmes. For that reason, the identical costs for the primary operation and related medication during hospital stay for the two programmes were not included in this analysis.

The loss of productivity was calculated from return to work rates.

### Sensitivity analysis

The calculated cost could be influenced by uncertainty regarding the concrete values for parameters, estimations and associations, because some of the parameters were deterministic point estimates with distribution and variety. This uncertainty has been quantified by a sensitivity analysis in order to identify the robustness of the results cost.

The effects of the two programmes were estimated using the quality of life assessments. Based on the patient reported quality of life measurement, a single index score (15D-score) on a 0–1 scale, representing the overall health-related quality of life, was calculated for each of the six time points for the two groups respectively by using a set of utility weights. The scores of the two groups were compared using area under curve and Mann-Whitney test. The level of significance was 0.05.

The analyses were performed using SPSS programme.

### Ethical considerations

The study was performed in accordance with the Helsinki II Declaration, and all patients participated after informed consent. The Regional Scientific Ethical Committee 01-041/03 approved the protocol, and the study has been registered in the international protocol registration system , ID NCT 00459966.

## Results

The integrated programme was 494€ less costly than the standard care programme regarding direct costs. The direct costs per patient were higher in the preoperative period due to the prehabilitation programme, but lower in the postoperative period due to shorter length of stay, see table 2.

**Table 2:** Pre- and postoperative differences in direct costs per patient randomised to the integrated programme, which included prehabilitation and early rehabilitation, and to the standard care program (in Euros)

	**Integrated programme**		**Standard care programme**	
	
	In total	Per patient	In total	Per patient
**Preoperative **introduction **I) Physiotherapist: **1 h × 28 patients × 27€* = **756€** **I) Physician support: **0.16 h × 5 patients × 34€* = **27€** **R) Nurse: **0.25 h × 32 patients × 28€* = **224€**	783	28	224	8

Training **I) Physiotherapist: **1/2 h × 28 patients × 27€* × 2 times = **27€**	756	27	0	0

Smoking intervention **I) Nurse: **2.8 h × 3 smokers × 28€* = **235€** **i) Equipments/medication: **65 € × 3 patients = **195€**	430	15	0	0

Alcohol intervention **I) Nurse: **2.8 h × 0 smokers × 28€* = **0€** **I) Equipments/medication: **27 € × 0 patients = **0€**	0	0	0	0

Optimised pain treatment **I) Physician: **0.25h × 28 patients × 34€* = **238€**	238	9	0	0

Preoperative costs		**79**		**8**

**Postoperative hospital **training **I) Physiotherapist: **1 h × 28 patients × 5 times × 27€* = **3,880€** **R) Physiotherapist: **1/2 h × 32 patients × 7 times × 27€*= **3,024€**	3,880	135	3,024	95

Pain treatment **I) Senior nurse: **0.16 h × 3 times × 28 patients × 30*€ =**403€** **I) Specialist: **0.16 h × 3 times × 28 patients × 61€* = **820€** **R) Senior nurse: **0.16 h × 3 times × 32 patients × 30*€ =**460€** **R) Specialist: **0.08 h × 3 times × 32 patients × 61€* = **468€**	1,223	44	928	29

Hospital stay **I) Bed-price: **164 € per day × 5 days × 28 patients = **22,960€** **R) Bed-price: **164 € per day × 7 days × 32 patients = **36,736€**	22,960	820	36,736	1,148

Second surgery **R) Stop bleeding: 8,247€**	0	0	8,247	258

Postoperative hospital costs per patient		**999**		**1,530**

**Postoperative primary care **GP **I) Contact: **43 contacts × 14€ per contact = **623€** **R) Contact: **61 contacts × 14€ per contact = **854€**	623	22	854	27

Emergency contacts **I) Contact: **3 contacts × 24€ per contact = **72€** **R) Contact: **10 contacts × 24€ per contact=**240€**	72	2	240	8

Physiotherapy **I) Private physiotherapist: **20 h × 45€*= 900€ **R) Private physiotherapist: **67 h × 45€*= 3,015€	900	32	3,015	94

Medical treatment **I) Medical treatment: 1120€** **R) Medical treatment: 32€**	1,120	40	32	1

Postoperative primary care costs per patients		**96**		**130**

**Balance for direct costs per patient**		**1,174**		**1,668**

* Salary per hour

There was a non-significant trend that the patients randomised to the integrated programme returned faster to work, 77 days (14–90), compared to the patients randomised to the standard care programme, 88 days (54–90); p = 0,092. The indirect costs as reported by the loss of productivity was correspondingly lower for the integrated programme; 8,021 € versus 9,152 €, when estimated by multiplication of the number of days and the Danish average salary (104€ per day).

The accumulated estimate of the differences in direct and indirect costs was 1,625€ (494€ + 1,131€) in favour of the integrated programme.

Regarding health related quality of life, there were no differences between the two groups when comparing the 15D-scores (table 3).

**Table 3:** Health related quality of life 15D-scores[8] for the patients randomised to the integrated program and the patients randomised to the standard care program

	**Integrated programme**	**Routine programme**
8 weeks preoperatively	0.83 (0.67–1.00)	0.79(0.63–0.95)
Operation day	0.85 (0.74–0.99)	0.82(0.65–.096)

Day of Discharge	0.83 (0.62–1.00)	0.79 (0.48–0.94)

1 month postoperatively	0.88 (0.74–0.99)	0.86 (0.66–0.96)

3 months postoperatively	0.90 (0.73–1.00)	0.89 (0.62–1.00)

6 months postoperatively	0.91 (0.73–1.00)	0.90 (0.69–1.00)

(Median and range)

The analysis of sensitivity included the best cases and worst cases for time consumption, salary, bed-cost, length of stay and return to work rate.

This analysis of the extreme values showed a variation in direct cost from 657€ to 3,239€ for the standard care programme and 553€ to 2532€ for integrated programme. The variation in total costs were 10,148€ to 15,906€ of the standard care programme and 8,117€ to 11,884€ for the integrated programme.

This analysis of the extreme values did not change the conclusion of the main base analysis, table 4.

**Table 4:** The variety of the pre- and postoperative costs in the two programmes reflected by the most optimistic and most pessimistic estimates (Euros)

	**Integrated programme**			**Routine programme**		
	
	Most optimistic	Base case	Most pessimistic	Most optimistic	Base case	Most pessimistic
Preoperative costs	22	79	88	5	8	9

Postoperative hospital costs	449	999	2,334	536	1,530	3,074

Postoperative primary care costs	82	96	111	116	130	157

Direct costs	553	1,174	2,532	657	1,668	3,239

Indirect costs	7,011	8,021	9,098	8,834	9,152	9,427

Balance	8,117	10,369	11,884	10,148	12,488	15,906

## Discussion

This study showed that prehabilitation supplemental to early rehabilitation after surgery of the lumbar spine is cost effective regarding direct hospital costs as well as indirect costs. The prehabilitation programme made the patient reach the postoperative milestones sooner, thus reducing the period of "inability" and thereby the length of stay significantly. The prehabilitation programme was not followed by more complications or pain.

This is the first time to evaluate the combination of prehabilitation and early rehabilitation after surgery of the lumbar spine. Previous studies of cost effectiveness of spine surgery had focused upon the surgical technique[9] or compared surgical intervention with non-surgical intervention[10].

The main effect of integrated programmes after surgery alone is a shorter hospitalization and reconvalescence based on better surgical and anaesthesiological techniques as well as patient-doctor agreements of faster rehabilitation. When comparing our length of stay to other studies, it is important to pay attention to possible difference regarding inclusion or exclusion of rehabilitation in the surgical department.

In the standard care programme the one case of revision surgery added costs of 258€/patient. As seen in table 2 this complication account for 15% of the extra costs in the standard care group. The complication may not be representative for the group and could be an occasional result. However, this patient had increased risk for complications due to smoking, which would have been preventable by the comprehensive prehabilitation programme. Even without this complication the costs of the standard care group would still be higher compared to the intervention group.

Integrated programmes have been shown to be cost effective for other surgical interventions than spine surgery; resection of colon[11], aorta surgery[12], lung resection[13], and orthopaedic surgery [14,15]

The prehabilitation programme did not change the complication rate and could therefore be interpreted as a waste of time and money. However, it did allow for an earlier return to work and decreased the amount of medical required post-operatively and the programme is therefore worthwhile given the economic benefits documented.

Evaluation of prehabilitation programmes of smoking cessation or alcohol intervention in standard surgical programmes showed a significant reduction in development of postoperative complications that required treatment [3,4]. Physical training programmes before hip- or knee replacement have shown contra-dictionary results which may however, be due to differences in intensity, duration and comprehensiveness of the evaluated training programmes [6,16].

When generalising the results of the present study the limitations should be considered. Our study is not generalisable owing to the country-specific differences in costs and policy. The difference of LOS in US an in Denmark is probably due to different routines regarding postoperative rehabilitation. The LOS in our study included rehabilitation at the surgical department. All patients was discharged directly to their home, nobody was transferred for or needed further physical therapy. The LOS of our control group corresponded to the LOS of recent studies, e.g. Elder JB and co-authors from Los Angeles, CA[17]. Their control and intervention groups stayed 6.96 vs. 6.36 days, respectively. Our LOS was 7 vs. 5 days. Another measurement could be physical therapy clearance for discharge 6.40 vs. 5.92 days reaching all milestones in our study; 6 vs. 4 days. A study of minimal invasive surgery presented a very short stay 3 and 4 days, but it did not include information on rehabilitation during the stay or after discharge from the surgical department[18].

The number of 60 patients was too small for detailed evaluation regarding types of complications, minor differences in quality of life and costs, which may all, have been overlooked due to a type-2 failure. Furthermore, the quality of life was assessed using the generic questionnaire 15D[8], which is reliable for comparison the life quality for patients suffering from different illnesses. It may, however, not be sensitive enough to identify differences between the two randomised groups.

A high compliance in the intervention group is essential for a proper evaluation of a beneficial effect on the outcome. In the present study the patients in the prehabilitation group were compliant to more than 80% of the training passes.

The estimations may be vitiated by different degrees of error regarding assessments, assumptions and associations. This is partly due to the use of deterministic point-estimates that are characterised by having no distribution or variety. The possible error seems limited in the present study, since the analysis of sensitivity based on both the most pessimistic and optimistic estimates did not change the conclusion, that a integrated programme of prehabilitation and early rehabilitation in spine surgery is more cost-effective than standard care, exclusively.

## Conclusion

This is the first study in this specific area of integrating prehabilitation and early rehabilitation after surgery of the lumbar spine and there is an inborn risk of type-1 failure, which can only be reduced by repeating the study. To overcome the potential type-2 failures the future studies should collect a sizeable number of data for the more detailed evaluation, and use a more specific questionnaire for assessment of quality of life in patients undergoing spine surgery.

## Competing interests

The authors declare that they have no competing interests.

## Authors' contributions

PRN designed and managed the study, recruited the patients and obtained, recorded, analyzed and interpreted the data. JA and MA analysed and interpreted the data. HT designed the study and analysed and interpreted the data. PRN and HT drafted the manuscript. JA and MA revised the manuscript critically. All authors read and approve the final manuscript.

## Pre-publication history

The pre-publication history for this paper can be accessed here:


